# Mangiferin inhibits multiple stages of the *Toxoplasma gondii* lytic cycle *in vitro* and protects against acute RH strain infection *in vivo*

**DOI:** 10.3389/fmicb.2026.1983878

**Published:** 2026-10-23

**Authors:** Shuoxiang Gao, Yingzhuo Dou, Mengyi Li, Junru Wu, Tingting Ying, Zhenchao Zhang, Zhenke Yang, Xuefang Mei, Shuai Wang, Xiaowei Tian

**Affiliations:** Department of Pathogenic Biology, School of Basic Medical Sciences, Henan Medical University, Xinxiang, China

**Keywords:** antiparasitic activity, mangiferin, natural product, RH strain, *Toxoplasma gondii*

## Abstract

**Introduction:**

Toxoplasmosis remains an important global health concern, highlighting the need for novel agents anti-*Toxoplasma gondii* (*T. gondii*). Mangiferin, derived from *Mangifera indica*, has demonstrated diverse pharmacological and antiprotozoal activities, but its effects against *T. gondii* remain unclear.

**Methods:**

We evaluated the anti-*T. gondii* activity of mangiferin *in vitro* and *in vivo*. Cytotoxicity toward human foreskin fibroblast (HFF) cells was assessed, followed by evaluation of parasite plaque formation and extracellular tachyzoite morphology. Its protective effects were further investigated in a murine model of acute RH strain infection, and its effects on parasite adhesion, invasion, and intracellular proliferation were examined.

**Results:**

Mangiferin showed low cytotoxicity toward HFF cells, with cell viability remaining above 90% at concentrations up to 320 μM. Mangiferin markedly inhibited parasite plaque formation and induced rapid morphological changes in extracellular tachyzoites. In a murine model of acute RH strain infection, mangiferin prolonged survival, alleviated splenic pathological damage, and reduced the relative parasite burden in the blood, spleen, and brain. Stage specific analyses further demonstrated that mangiferin inhibited parasite adhesion, invasion, and intracellular proliferation.

**Conclusion:**

Mangiferin exhibits anti-*T. gondii* activity both *in vitro* and *in vivo* and affects multiple processes within the *T. gondii* lytic cycle. These findings provide initial experimental evidence supporting further investigation of mangiferin as a potential natural compound for the development of novel strategies against toxoplasmosis.

## 1 Introduction

*Toxoplasma gondii* (*T. gondii*) is an obligate intracellular protozoan parasite of the phylum Apicomplexa that can infect nearly all warm-blooded animals, including humans. Toxoplasmosis is a globally prevalent zoonotic disease that poses a substantial threat to human health and animal production (De Barros et al., 2022). Most immunocompetent individuals experience asymptomatic or mild *T. gondii* infection; however, severe and potentially fatal disease may develop in immunocompromised patients and during pregnancy (Matta et al., 2021; Aerts et al., 2024).

Despite the availability of established therapies, pyrimethamine based regimens, particularly pyrimethamine combined with sulfadiazine, remain a cornerstone of treatment for clinically significant toxoplasmosis. However, their clinical use is limited by substantial toxicity, with bone marrow suppression being one of the major treatment-related adverse effects. A systematic review of 31 studies involving 2,975 patients found that more than half of the studies reported treatment discontinuation or modification because of adverse events, with hematological, gastrointestinal, and dermatological toxicities being among the most frequently reported complications (Ben-Harari et al., 2017). These safety concerns are particularly relevant in vulnerable populations, including pregnant women and immunocompromised patients, in whom treatment options may be further constrained by potential maternal, fetal, or treatment-related toxicity (Walana et al., 2026). During pregnancy, therapeutic strategies are also dependent on gestational age and evidence of fetal infection, further complicating clinical management (Ribeiro et al., 2025; Gomes Ferrari Strang et al., 2023). Collectively, the limitations of current anti-*T. gondii* therapies highlight the importance of exploring additional therapeutic candidates.

Natural products represent an important source of bioactive compounds for the discovery of novel antiparasitic agents. In particular, medicinal plants have attracted increasing attention because of their diverse phytochemical constituents and broad pharmacological activities (Shang X. et al., 2025). *Mangifera indica* (*M. indica*), commonly known as mango, has a long history of medicinal use, and extracts and bioactive constituents from this plant have been reported to exhibit a broad spectrum of biological activities, including antibacterial, antiviral, anti-inflammatory, and antiparasitic effects (Mendonça et al., 2025). Among these constituents, mangiferin, a naturally occurring C-glucosylxanthone, has attracted considerable attention owing to its diverse pharmacological activities and demonstrated antiparasitic potential (Zivković et al., 2024). Notably, mangiferin has shown inhibitory activity against *Plasmodium* spp., providing direct evidence of its antiprotozoal activity (Maenpuen et al., 2023).

Despite the diverse pharmacological activities and reported antiparasitic potential of mangiferin, its activity against *T. gondii* remains unclear. Given the limitations of currently available therapies for toxoplasmosis, investigating the anti-*T. gondii* activity of mangiferin may help identify potential candidates for further drug development. Therefore, the present study evaluated the effects of mangiferin on *T. gondii* using complementary *in vitro* and *in vivo* approaches and further characterized its effects on key stages of the parasite lytic cycle. The findings provide experimental evidence for the anti-*T. gondii* activity of mangiferin and a basis for further investigation of its therapeutic potential.

## 2 Materials and methods

### 2.1 Animal ethics statement

All animal studies in this study were performed following the ethical guidelines of the Animal Ethics Committee of Henan Medical University and were approved under protocol number XYLL-20220223. Mice reaching humane endpoints, as indicated by obvious clinical signs during survival observations, or those at the predefined experimental endpoint on day 7 post infection were anesthetized by inhalation of isoflurane (5% in oxygen) to achieve deep anesthesia, as indicated by the complete absence of reflex responses. The animals were then euthanized by cervical dislocation, and death was verified by the absence of respiration and heartbeat. Appropriate measures were taken throughout the experiments to minimize animal discomfort and suffering.

### 2.2 Mice, cells and parasites

Six to Eight weeks old female BALB/c mice purchased from SPF (Beijing) Biotechnology Co., Ltd. were maintained under normal conditions without any specific pathogen. The *T. gondii* RH strain was stored at the Pathogenic Biology Department at Henan Medical University. As previously described, the RH strain was maintained in human foreskin fibroblasts (HFF; ATCC, Catalog No. SCRC-1041™) (Wan et al., 2023). HFF cells were maintained in Dulbecco’s Modified Eagle Medium (DMEM) containing 2% fetal bovine serum (FBS), with both products purchased from Procell, China.

### 2.3 Cytotoxicity of mangiferin on HFF cells

HFF cells were plated into 96-well plates at 4 × 10^3^ cells/well in 100 μL of complete DMEM and incubated for 12 h to allow cell attachment. Cells were then treated with mangiferin (purity ≥ 95%, Shanghai Aladdin Biochemical Technology Co., Ltd., China) at concentrations ranging from 10 to 2,560 μM (10, 20, 40, 80, 160, 320, 640, 1,280, and 2,560 μM). A DMSO vehicle control, medium control, and blank control without cells were included. After 48 h of treatment, 10 μL of CCK-8 solution was added to each well and incubated for 2 h. Cell viability was determined by measuring the absorbance at 450 nm using a microplate reader (Thermo Scientific, United States).

### 2.4 Plaque assay

6-well plates containing fully confluent HFF cells were infected with 200 freshly egressed RH tachyzoites per well and incubated undisturbed for 6–7 days at 37 °C with 5% CO₂ in the presence of 160 μM or 320 μM mangiferin. DMSO control group (diluted 1:1000) and a sulfadiazine (SD) positive control group (100 μg/mL) were included in parallel. Upon plaque formation, the cultures underwent fixation in 4% paraformaldehyde for 15 min and were subsequently subjected to crystal violet staining (0.5%) for 60 min. The plates were then scanned (Canon, Japan) for subsequent plaque analysis. The number and area of plaques were measured using standardized ImageJ-based analysis procedure. The same analysis criteria were applied to all experimental groups. When no detectable plaques were identified, the plaque count and detectable plaque area were both recorded as zero. The experiment was independently repeated three times.

### 2.5 Morphological assays

#### 2.5.1 Quantification of tachyzoite rounding

Freshly egressed *T. gondii* tachyzoites were collected and aliquoted into 24-well plates at 5 × 10^5^ tachyzoites per well. Tachyzoites were treated with DMSO or mangiferin at 160 or 320 μM for 30 min, 1 h, 2 h, or 4 h. Independent wells were prepared for each treatment concentration and time point. At the indicated time points, parasite morphology was examined under a light microscope at 40 × magnification. Multiple microscopic fields were randomly selected for each well, and at least 100 tachyzoites were counted per well. Tachyzoites exhibiting a distinct rounded morphology with loss of their characteristic crescent shaped appearance were classified as rounded parasites. Morphological assessment was performed in a blinded manner. The percentage of rounded tachyzoites was calculated as the number of rounded parasites divided by the total number of tachyzoites counted × 100%.

#### 2.5.2 Scanning electron microscopy

Freshly egressed *T. gondii* tachyzoites were treated with 320 μM mangiferin for 2 h, with DMSO-treated parasites serving as the control. Following treatment, the parasites were collected by centrifugation and fixed with 2.5% glutaraldehyde. After fixation, the samples were rinsed three times with distilled water for 10 min each and dehydrated through a graded ethanol series (30%, 50%, 70%, and 90%), followed by two changes of absolute ethanol. The samples were then dried using a supercritical point dryer (Shianjia Technology, China), mounted on specimen stubs with conductive carbon adhesive tape, and sputter-coated with gold using an ion sputter coater (SuPro Instruments, China). The prepared samples were examined using a scanning electron microscope (CIQTEK, China). Representative micrographs were acquired at appropriate magnifications to assess overall parasite morphology and surface features.

### 2.6 Animal experiment

#### 2.6.1 *Toxoplasma gondii* infection and mangiferin treatment

Female BALB/c mice aged 6–8 weeks were randomly divided into four groups, with 14 mice per group: a sodium carboxymethyl cellulose (CMC-Na) vehicle control group, a 50 mg/kg mangiferin group, a 100 mg/kg mangiferin group, and a 200 mg/kg mangiferin group. Mice were intraperitoneally infected with 100 RH tachyzoites to establish acute toxoplasmosis. Four hours post infection, mangiferin or vehicle was administered intragastrically once daily for 7 consecutive days.

#### 2.6.2 Evaluation of the effects of mangiferin against acute RH strain infection

Ten mice from each group were monitored daily for survival throughout the experiment, and survival curves were generated. Four mice from each group were euthanized on day 7 post infection for subsequent analyses. Body weight and spleen weight were recorded, and the spleen index was calculated as follows: spleen index (%) = spleen weight/body weight × 100%. Spleen tissues were paraffin embedded, sectioned, and stained with hematoxylin and eosin (HE) for histopathological examination (*n* = 4).

#### 2.6.3 Quantification of tissue parasite burden by qPCR

Peripheral blood samples were collected after red blood cell lysis, and brain tissues were harvested. The parasite burden was quantified by qPCR using *SAG1* as the target gene and mouse *Actin* (M-*Actin*) as the reference gene (*n* = 4). Primer sequences used for qPCR are summarized in Supplementary Table S1.

### 2.7 *In vitro* anti-*toxoplasma* activity

#### 2.7.1 Adhesion assay

24-well plates containing confluent HFF monolayers were fixed with 4% paraformaldehyde before the adhesion assay. The cells were then incubated with 5 × 10^5^ freshly egressed RH tachyzoites per well in the presence of the indicated concentrations of mangiferin or vehicle control at 37 °C for 30 min. After incubation, cells were gently washed three times with precooled PBS to remove unbound parasites. The number of tachyzoites attached to the cell surface was counted in 10 randomly selected fields per coverslip under an inverted microscope at 40 × magnification. The experiment was independently repeated three times.

#### 2.7.2 Invasion assay

HFF monolayers at approximately 80% confluency in 24-well plates were exposed to 5 × 10^5^ freshly released RH tachyzoites per well. Tachyzoites were prelabelled with CFDA-SE (Glpbio, United States). Following a 2 h incubation, cells were washed to remove extracellular tachyzoites, and then cultured in medium containing the indicated concentrations of mangiferin or vehicle control. After 12 h, cells were then harvested by trypsinization, and FITC positive HFF cells were analyzed via flow cytometry (Beckman, United States). The experiment was independently repeated three times.

#### 2.7.3 Intracellular proliferation assay

Upon reaching approximately 80% confluency, HFF monolayers in 12-well plates were inoculated with 1 × 10^6^ egressed RH tachyzoites freshly released from HFF. Following a 2 h incubation, cells were washed to remove extracellular tachyzoites, and then cultured in medium containing the indicated concentrations of mangiferin or vehicle control. Cells were harvested after continuous drug treatment for 12 h, 24 h, and 48 h. Intracellular *T. gondii* proliferation was quantified by qPCR using *SAG1* as the target gene and human *Actin* (H-*Actin)* as the reference gene according to the 2^-∆∆ct^ method. The experiment was independently repeated three times. Primer sequences used for qPCR are summarized in Supplementary Table S1.

### 2.8 Statistical analysis

Statistical analyses were conducted with GraphPad Prism 8.0. Data from multiple groups were analyzed by one way analysis of variance, followed by Tukey’s or Dunnett’s multiple comparisons test, as appropriate. Plaque assay were analyzed using the Kruskal-Wallis test followed by Dunn’s multiple-comparison test. Survival curves were evaluated using the Kaplan–Meier method and compared by the log-rank test. *p* value < 0.05 was considered statistically significant. Statistical significance was denoted as follows: *p* < 0.05 (*), *p* < 0.01 (**), *p* < 0.001 (***), *p* < 0.0001 (****), and *p* ≥ 0.05 (ns).

## 3 Results

### 3.1 Mangiferin exhibits low cytotoxicity toward HFF cells

Mangiferin is a major C-glucosyl xanthone derived from *M. indica*, and its chemical structure is shown in Figure 1A. Before evaluating its anti-*T. gondii* activity, the cytotoxicity of mangiferin toward HFF cells was evaluated using a CCK-8 assay following treatment with increasing concentrations of mangiferin. Cell viability remained above 90% at concentrations up to 320 μM, whereas concentrations exceeding 320 μM resulted in a reduction in cell viability to below 90%. Accordingly, 160 and 320 μM mangiferin were selected for subsequent *in vitro* experiments (Figure 1B).

**Figure 1:**
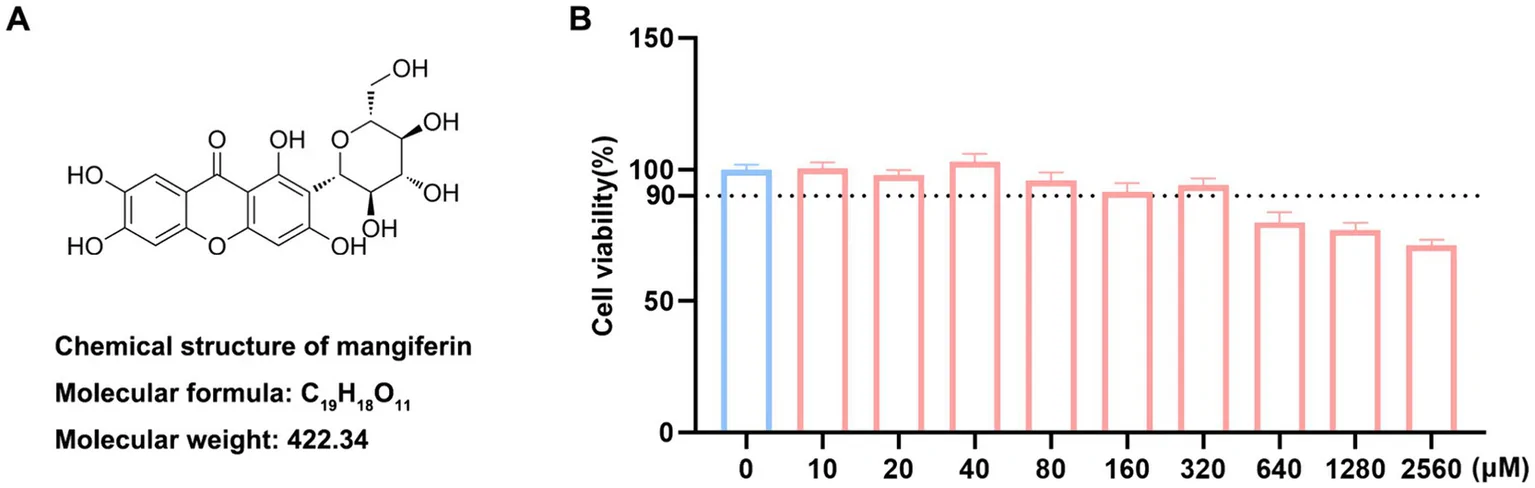
Cytotoxicity of mangiferin in HFF cells. **(A)** Chemical structure of mangiferin. **(B)** Cell viability was determined by CCK-8 assay after 48 h of mangiferin treatment. Data are presented as mean ± SD.

### 3.2 Mangiferin inhibits *Toxoplasma gondii* growth

To investigate the effect of mangiferin on the lytic growth of *T. gondii*, a plaque assay was performed in HFF monolayers. Compared with the DMSO control, 160 μM mangiferin reduced plaque numbers, although the difference was not statistically significant, whereas no plaques were detected in the 320 μM group (Figures 2A,B). The Kruskal-Wallis test revealed a significant overall difference among groups (*p* = 0.0006). Dunn’s multiple-comparison test identified a significant difference only between the DMSO control and the 320 μM group (*p* < 0.05); all other pairwise comparisons were not significant. A similar pattern was observed for relative plaque area (Figure 2C). Treatment with 160 μM mangiferin reduced the relative plaque area compared with the DMSO control, but the difference was not statistically significant. No detectable plaque area was observed in the 320 μM group. The overall difference among groups was significant (Kruskal-Wallis test, *p* = 0.0013), with Dunn’s multiple-comparison test showing a significant difference only between the DMSO control and the 320 μM group (*p* < 0.05).

**Figure 2:**
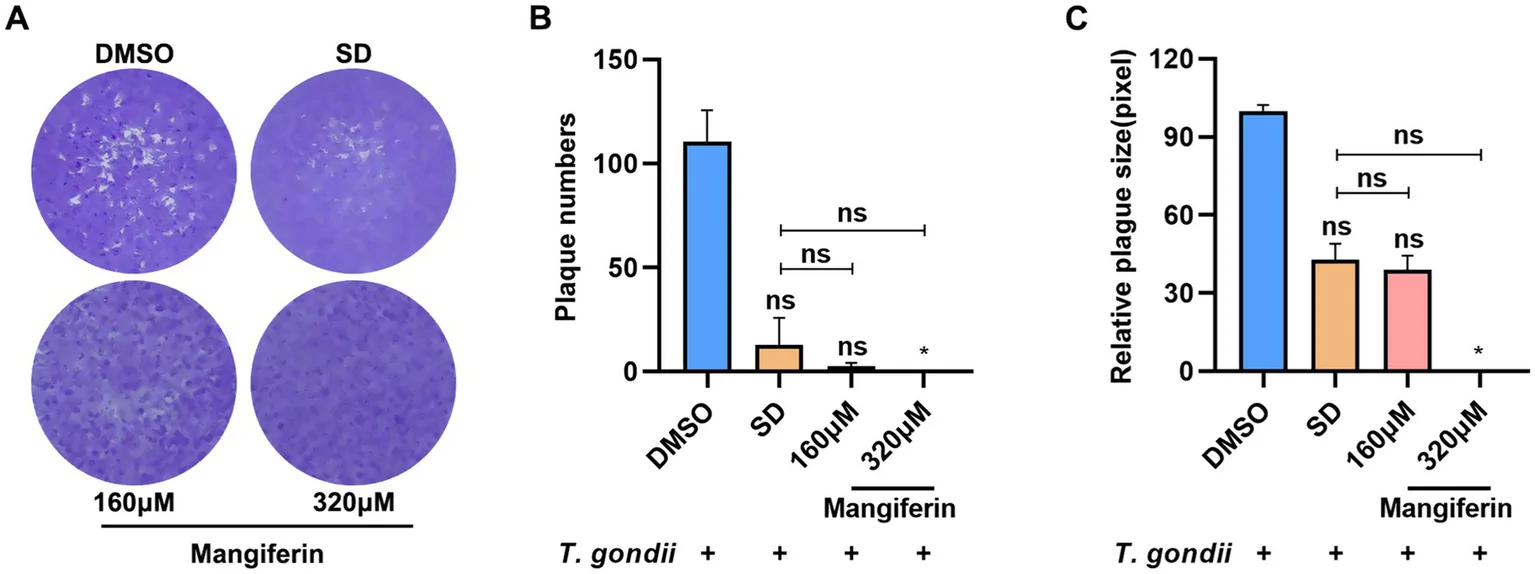
Mangiferin inhibits plaque formation in HFF cells and induces morphological changes in *T. gondii*. **(A)** Representative images of *T. gondii* plaques following treatment with mangiferin or controls. **(B)** Quantification of plaque numbers in each treatment group. **(C)** Quantification of the relative plaque area. Data are presented as mean ± SD.

### 3.3 Mangiferin induces morphological alterations in *Toxoplasma gondii* tachyzoites

In addition to inhibiting parasite growth, mangiferin affected the morphology of *T. gondii* tachyzoites. The proportion of rounded parasites increased following mangiferin treatment, with this effect becoming evident as early as 30 min and more pronounced with prolonged exposure, particularly at 320 μM (Figures 3A–D).

**Figure 3:**
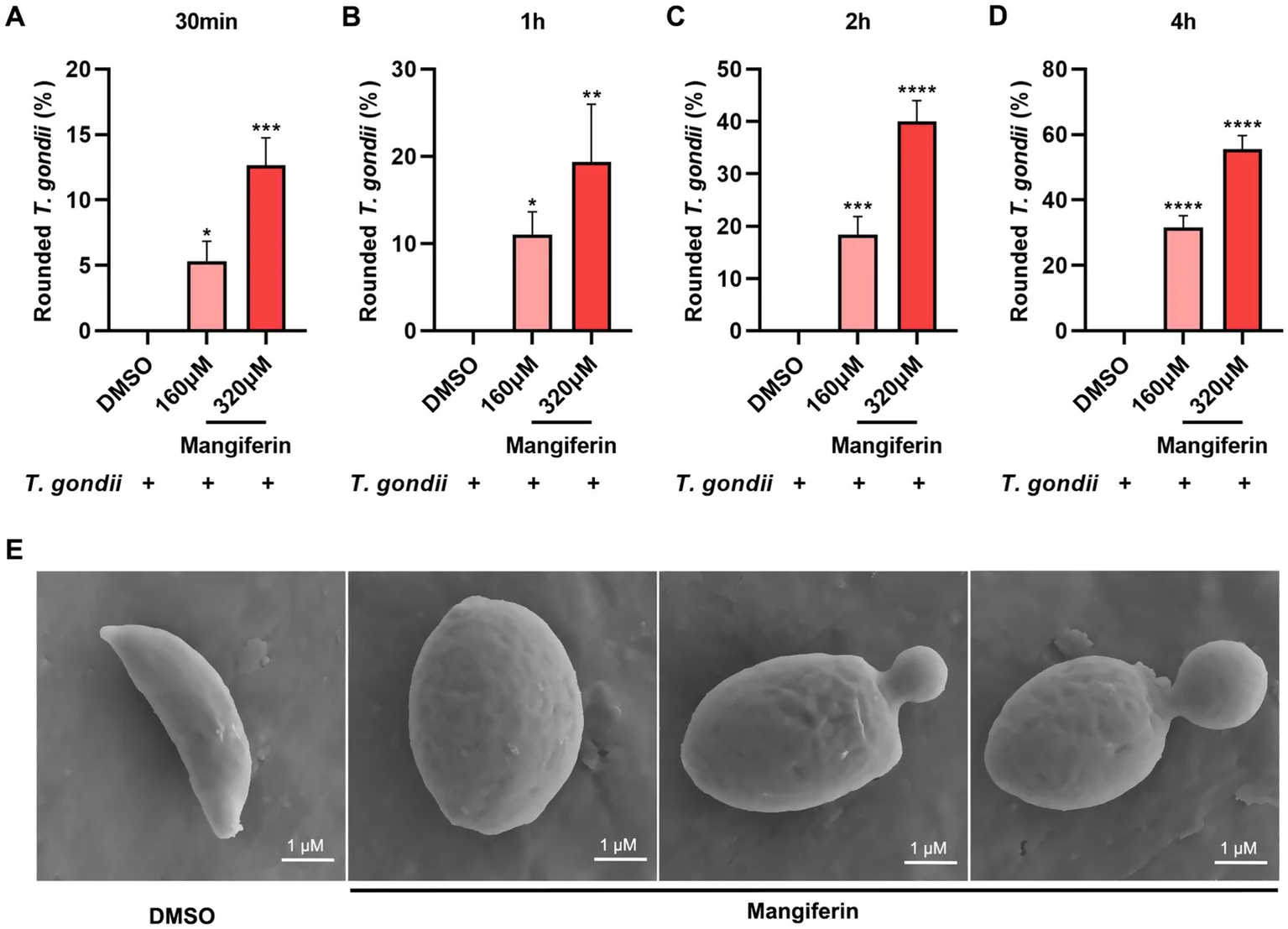
Mangiferin induces morphological changes in *T. gondii*. **(A–D)** Quantification of the proportion of rounded *T. gondii* following treatment with mangiferin for 30 min **(A)**, 1 h **(B)**, 2 h **(C)**, and 4 h **(D)**, respectively. Data are presented as mean ± SD. **(E)** Representative images of RH strain tachyzoites following treatment with mangiferin or DMSO.

To further characterize the morphological alterations induced by mangiferin, SEM was performed. Scanning electron microscopy revealed morphological alterations in *T. gondii* tachyzoites following treatment with 320 μM mangiferin for 2 h. Compared with the DMSO control group, some treated tachyzoites exhibited a rounded morphology, accompanied by localized surface protrusions and irregularities (Figure 3E).

### 3.4 Mangiferin improves survival and alleviates splenic pathology in mice with acute RH strain infection

Given the inhibitory effect of mangiferin on the lytic growth of *T. gondii* observed *in vitro*, its protective efficacy was next evaluated in a murine model of acute RH strain infection by monitoring survival and pathological changes following treatment with different doses of mangiferin. All mice in the CMC-Na control group died by day 7 post infection. In the 50 and 100 mg/kg mangiferin groups, survival was observed until day 8 post infection, with no significant difference in survival duration compared with the CMC-Na group. In contrast, mice receiving 200 mg/kg mangiferin remained alive until day 15 post infection, indicating a significant delay relative to the CMC-Na group (*p* < 0.01; Figure 4A).

**Figure 4:**
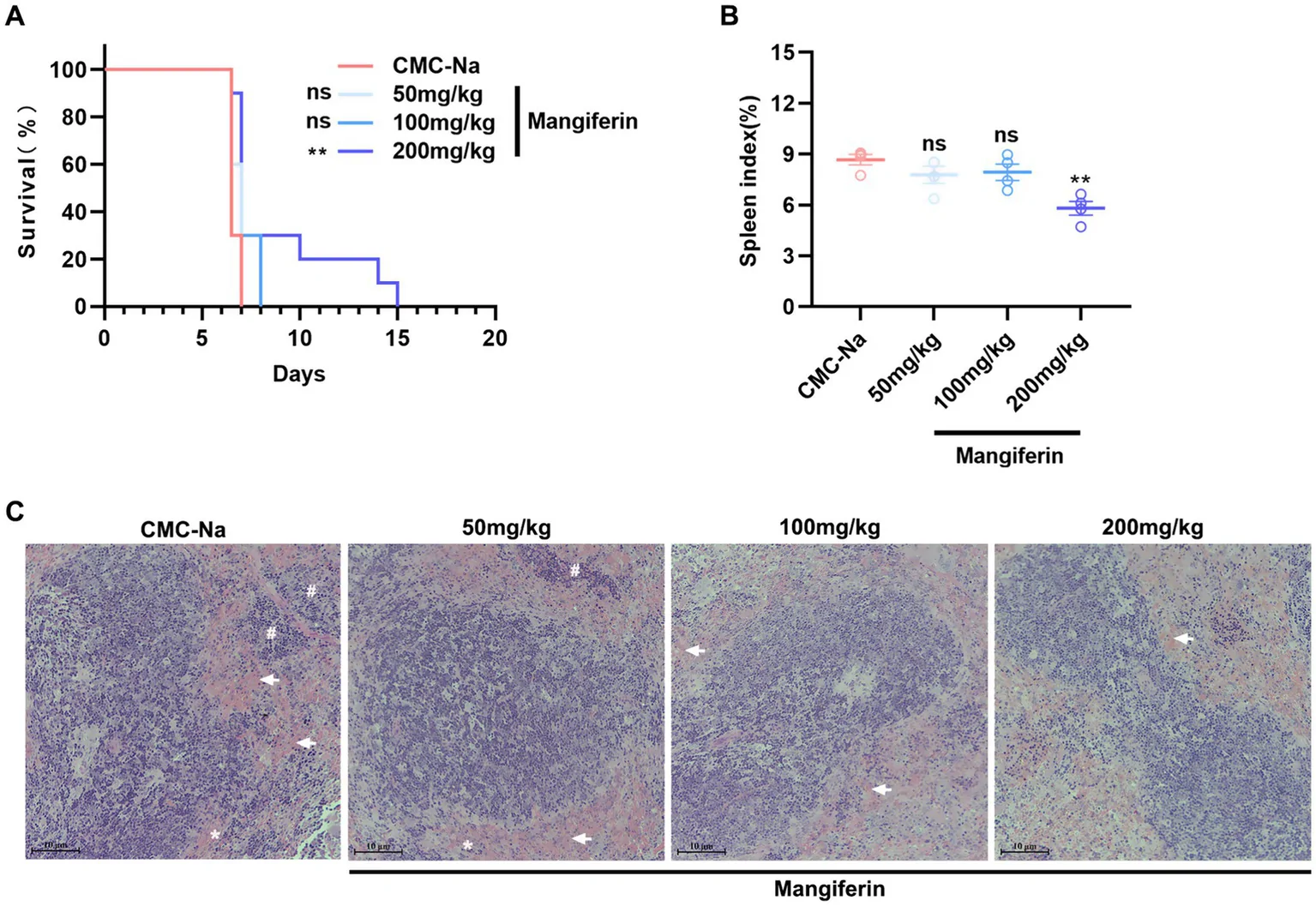
Protective effects of mangiferin against acute *T. gondii* infection. **(A)** Kaplan–Meier survival curves of infected mice treated with different doses of mangiferin. **(B)** Spleen index of mice on day 7 post infection. **(C)** Representative HE stained images of spleen tissues. Data are presented as mean ± SEM. Scale bar = 10 μm. Arrows indicate red pulp congestion, # indicates lymphocytic infiltration, and * indicates poorly defined boundaries between the white pulp and red pulp.

Spleen related pathological changes were subsequently assessed in mice euthanized on day 7 post infection. The spleen index differed significantly among the four groups [one-way ANOVA, *F* (3, 12) = 7.998, *p* = 0.0034, *R*^2^ = 0.667; Figure 4B]. Dunnett’s multiple-comparisons test showed that the spleen index was significantly lower in the 200 mg/kg mangiferin group than in the CMC-Na group (mean difference = 2.868 percentage points, 95% CI: 1.224–4.511 percentage points, *p* = 0.0014). No statistically significant differences were detected between the CMC-Na group and the 50 or 100 mg/kg mangiferin groups (*p* = 0.3606 and 0.5083, respectively). Histopathological examination revealed marked disruption of splenic architecture following *T. gondii* infection, characterized by extensive lymphocytic infiltration, poorly defined boundaries between the white and red pulp, and red pulp congestion. Mangiferin treatment alleviated these pathological alterations and partially preserved the splenic architecture. Notably, the splenic architecture was relatively well preserved in mice receiving 200 mg/kg mangiferin (Figure 4C). Collectively, these findings provide preliminary evidence of a protective effect of mangiferin, particularly at 200 mg/kg, against acute *T. gondii* infection, as reflected by prolonged survival and reduced splenic pathological changes.

### 3.5 Mangiferin reduces *Toxoplasma gondii* burden in infected mice

To further determine whether the protective effects of mangiferin were associated with reduced parasite replication *in vivo*, the relative *T. gondii* burden in the blood, spleen, and brain was quantified on day 7 post infection. One-way ANOVA revealed significant differences among the four groups in blood [*F* (3, 12) = 30.47], spleen [*F* (3, 12) = 71.86], and brain [*F* (3, 12) = 53.85] (all *p* < 0.0001). Dunnett’s multiple-comparisons tests showed significantly lower relative parasite levels in all three mangiferin-treated groups than in the CMC-Na group across all three tissues (all adjusted *p* < 0.0001; Figures 5A–C).

**Figure 5:**
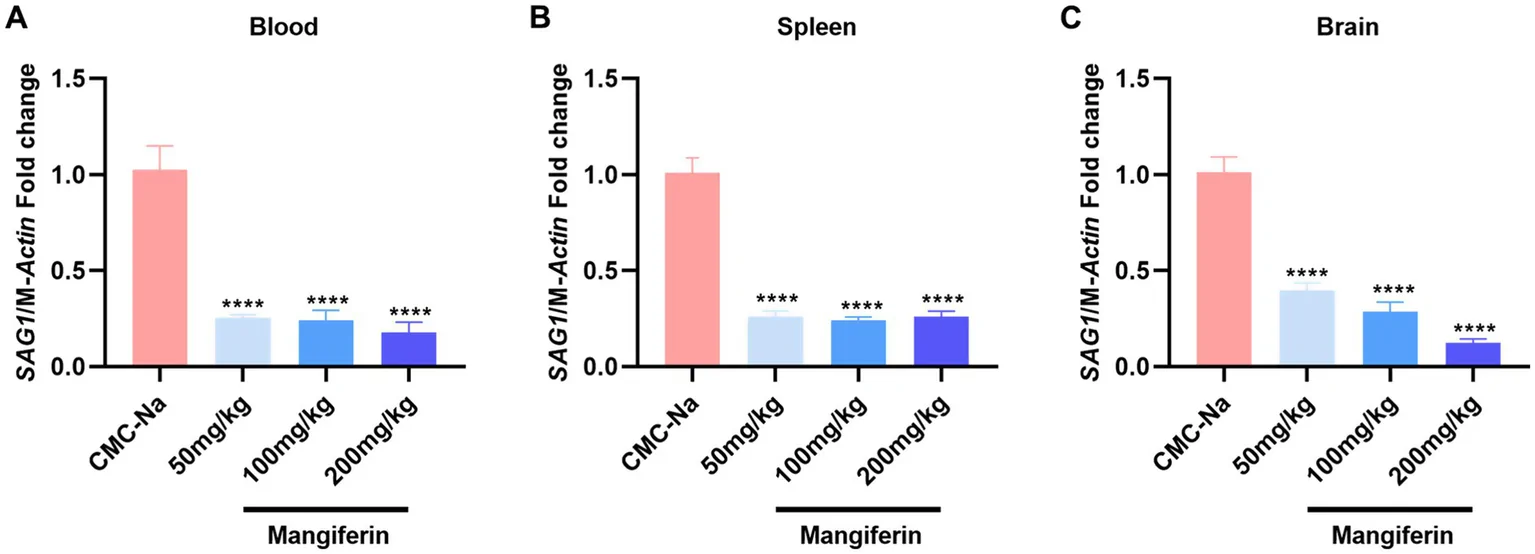
Mangiferin reduces *T. gondii* burden in infected mice. Relative *T. gondii* burden in the blood **(A)**, spleen **(B)**, and brain **(C)** was quantified by qPCR on day 7 post infection. Data are presented as mean ± SEM.

### 3.6 Effects of mangiferin on different stages of parasite infection

To clarify the stage of the *T. gondii* life cycle targeted by mangiferin, its effects on parasite adhesion, invasion, and intracellular proliferation in HFF cells were subsequently evaluated. In the adhesion assay, compared with the DMSO control group, SD treatment did not significantly affect the number of *T. gondii* tachyzoites adhering to HFF cells (ns). In contrast, treatment with either 160 μM or 320 μM mangiferin significantly reduced parasite adhesion (*p* < 0.0001), as shown in Figure 6A. Consistently, the invasion assay showed that SD treatment did not significantly alter the proportion of CFDA-SE^+^ HFF cells compared with the DMSO control group (ns), whereas treatment with either 160 μM or 320 μM mangiferin significantly reduced the proportion of CFDA-SE^+^ HFF cells (*p* < 0.0001; Figure 6B), indicating an inhibitory effect of mangiferin on parasite invasion.

**Figure 6:**
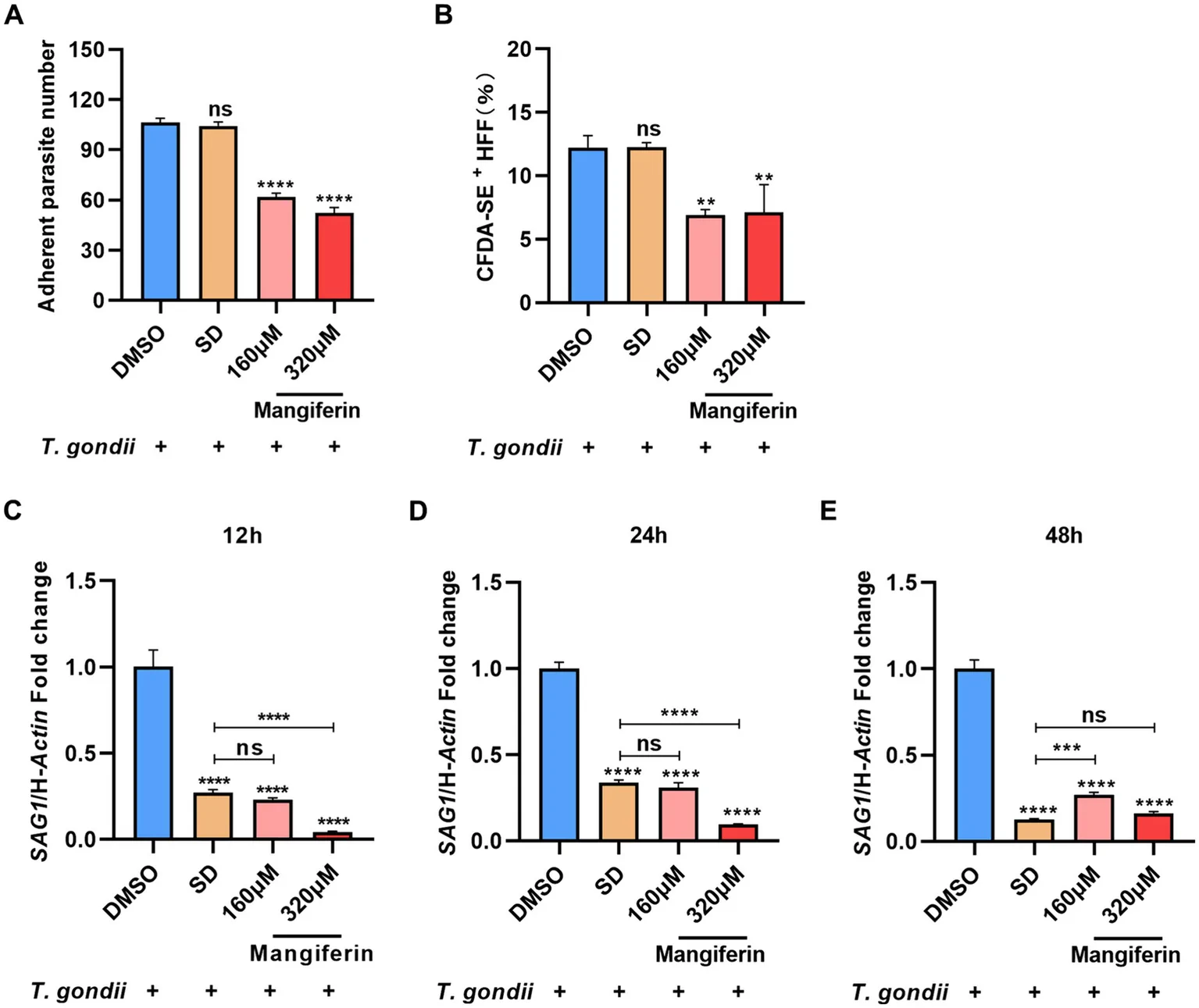
Effects of mangiferin on *T. gondii* adhesion, invasion, and intracellular proliferation in HFF cells. **(A)** Effects of mangiferin on the adhesion of *T. gondii* tachyzoites to HFF cells. **(B)** Effects of mangiferin on *T. gondii* invasion of HFF cells. **(C–E)** Effects of mangiferin on intracellular proliferation of *T. gondii* at 12 **(C)**, 24 **(D)**, and 48 h **(E)** post treatment. Data are presented as mean ± SD.

The effect of mangiferin on intracellular proliferation of *T. gondii* was subsequently evaluated at 12, 24, and 48 h post treatment. At both 12 and 24 h, treatment with 160 μM or 320 μM mangiferin significantly inhibited intracellular parasite proliferation compared with the DMSO control group (both *p* < 0.0001). Notably, the inhibitory effect of 320 μM mangiferin was significantly greater than that of SD at these time points (*p* < 0.0001; Figures 6C,D). At 48 h, mangiferin continued to significantly suppress intracellular parasite proliferation (*p* < 0.0001); however, no significant difference was observed between the 320 μM mangiferin and SD groups (ns), indicating comparable inhibitory effects at this time point (Figure 6E).

## 4 Discussion

Toxoplasmosis remains an important global health concern, and the limitations of currently available therapies highlight the need for novel anti-*T. gondii* agents (Liu et al., 2024). In the present study, mangiferin demonstrated anti-*T. gondii* activity in both *in vitro* and *in vivo* models. Notably, its activity extended beyond overall lytic growth inhibition to multiple parasites associated processes, including adhesion, invasion, and intracellular proliferation, thereby providing initial experimental evidence supporting the anti-*T. gondii* potential of mangiferin.

The relatively low cytotoxicity previously reported for mangiferin provides an important basis for interpreting its antiparasitic activity (Mei et al., 2023; Yehia and Altwaim, 2023). Consistent with these observations, HFF cell viability remained above 90% following treatment with mangiferin at concentrations up to 320 μM in the present study, supporting the use of this concentration range for subsequent evaluation of its anti-*T. gondii* activity. Importantly, marked inhibition of *T. gondii* growth was observed at concentrations that caused no substantial loss of HFF cell viability, indicating that the antiparasitic phenotype was not simply attributable to overt host-cell cytotoxicity.

The inhibitory effect of mangiferin on *T. gondii* lytic growth was first demonstrated by the plaque assay. Plaque formation reflects successive rounds of host-cell invasion, intracellular replication, egress, and reinvasion, and thus provides an integrated measure of parasite lytic growth (Ufermann et al., 2017). In the present study, mangiferin markedly reduced plaque formation in HFF monolayers, supporting an inhibitory effect on the overall parasite lytic cycle. In parallel, mangiferin induced a rapid increase in the proportion of rounded extracellular tachyzoites. This phenotype was detectable within 30 min of treatment and became more pronounced with increasing exposure time and mangiferin concentration, suggesting an early morphological response of extracellular tachyzoites to mangiferin exposure. Consistent with these findings, SEM revealed a rounded morphology in some *T. gondii* tachyzoites following treatment with 320 μM mangiferin for 2 h. In addition, some rounded tachyzoites exhibited localized, spherical surface protrusions of varying apparent sizes. Similar morphological abnormalities, including rounding and deformation of tachyzoites, have been reported following exposure to various anti-*Toxoplasma* compounds and are often accompanied by impaired parasite growth, invasion, or structural abnormalities (De Oliveira et al., 2025; Guo et al., 2025; Teixeira et al., 2025). These findings suggest that the morphological changes observed following mangiferin treatment may be associated with its inhibitory effects on parasite growth. However, the structural nature and biological significance of the observed surface protrusions remain unclear and warrant further investigation.

The antiparasitic activity of mangiferin observed *in vitro* was further supported in a murine model of acute RH strain infection. Mangiferin treatment prolonged the survival of infected mice and alleviated splenic pathological alterations, with the most pronounced protective effects observed at 200 mg/kg. In parallel, mangiferin treatment significantly decreased the relative *T. gondii* burden in the blood, spleen, and brain, providing *in vivo* evidence consistent with the antiparasitic activity observed *in vitro*. Notably, the dose-dependent effects on survival did not fully correspond to the reductions in relative *T. gondii* burden. Previous studies have demonstrated that disease outcomes following *T. gondii* infection are influenced not only by parasite burden but also by host immune responses and inflammation associated tissue damage. For example, Khan et al. reported that, in *T. gondii* infected C57BL/6 mice, early tissue parasite burdens were largely comparable between CCR5 deficient and wild type mice, despite differences in survival associated with the magnitude of the inflammatory response and immune-mediated tissue injury (Khan et al., 2006). Similarly, intestinal pathology during acute toxoplasmosis has been shown to depend on IL-4 mediated immune regulation and to be dissociated from parasite burden (Nickdel et al., 2004). These findings highlight the complex relationship between parasite burden, host immune responses, and disease outcome. Therefore, further studies combining parasite burden measurements with assessments of host immune responses and inflammation associated tissue injury are warranted to clarify the observed discrepancy between reductions in parasite burden and survival outcomes. In addition, further studies incorporating uninfected mice treated with mangiferin are needed to determine whether mangiferin itself affects host health in the absence of infection, thereby helping to distinguish its effects on the host from those associated with the response to infection. Although the present findings suggest protective effects of mangiferin, the relatively small sample size may limit the precision and robustness of the results. Further studies with larger sample sizes and appropriate experimental designs are warranted to validate these findings and further characterize the dose-dependent effects of mangiferin *in vivo*.

Another point worth noting is the difference between the *in vitro* activity and *in vivo* efficacy of mangiferin. In the present mouse model, mangiferin was administered orally, and its effects may therefore have been influenced by its absorption, metabolism, and tissue distribution. Previous studies have reported relatively low oral bioavailability of mangiferin (Mei et al., 2023; Wang et al., 2013), which may be one factor influencing it’s *in vivo* efficacy, although its actual impact in the present model remains to be determined.

Successful invasion of *T. gondii* into host cells requires the coordinated execution of parasite motility, attachment, secretion of invasion associated factors, and establishment of the parasitophorous vacuole (Carruthers and Sibley, 1997; Haase et al., 2025). Therefore, following the demonstration of anti-*T. gondii* activity of mangiferin against the RH strain *in vitro* and *in vivo*, its effects on different stages of the parasite lytic cycle were further examined to gain preliminary insight into the processes underlying its antiparasitic activity. Mangiferin significantly inhibited parasite adhesion and invasion, indicating that its activity extends to the early stages of parasite–host cell interaction, while the sustained inhibition of intracellular proliferation suggests that its inhibitory effect is maintained after parasite entry into host cells. Notably, the phenotypic activity profile of mangiferin differed from that of SD. SD primarily inhibits dihydropteroate synthase, thereby disrupting folate biosynthesis and ultimately impairing nucleotide synthesis and parasite proliferation (Hopper et al., 2019). The relatively weak effects of SD on parasite adhesion and invasion observed in the present study are consistent with previous observations showing that sulfadiazine exerts only a limited effect on tachyzoite invasion (Li et al., 2020). In contrast, mangiferin affected both early parasite–host cell interactions and intracellular proliferation, and exerted a stronger inhibitory effect on intracellular proliferation than SD at earlier time points, whereas the two treatments showed comparable effects at 48 h. These findings indicate that, under the *in vitro* conditions examined, mangiferin affects a broader range of parasite-associated processes than SD. The distinct activity profiles of the two compounds raise the possibility of complementary antiparasitic effects when used in combination.

Although the above findings demonstrate the inhibitory effects of mangiferin on multiple parasite-associated processes, the molecular basis underlying these effects remains unclear. Evidence from other protozoan parasites suggests that mangiferin may affect parasite metabolic enzymes. For example, mangiferin has been reported to inhibit serine hydroxymethyltransferase in *Plasmodium falciparum* (Maenpuen et al., 2023). However, whether this or other molecular targets contribute to the anti-*T. gondii* activity of mangiferin remains to be determined. In addition, indirect antiparasitic effects mediated through alterations in host-cell physiology cannot be completely excluded. Previous studies have shown that mangiferin can modulate multiple host-related signaling pathways, including NF-κB, NLRP3, MAPK, JAK/STAT, and Nrf2, and can influence cellular processes associated with oxidative stress and autophagy (Shang Y. et al., 2025; Jayasuriya et al., 2024; Feng et al., 2022; Gendy et al., 2022; Liu et al., 2025). These activities raise the possibility that host-cell responses may also contribute to the observed antiparasitic effects, although this possibility remains to be investigated. Nevertheless, the low cytotoxicity of mangiferin toward HFF cells, its inhibitory effects on multiple parasite-associated processes, and its protective activity in an acute RH strain infection model support its potential as a candidate anti-*T. gondii* compound. Importantly, given the distinct biological characteristics of different *T. gondii* strains, future studies using additional strains are warranted to evaluate the broader applicability of mangiferin. Further mechanistic studies are also needed to clarify the molecular basis underlying its anti-*T. gondii* activity.

Taken together, this study provides initial evidence that mangiferin exhibits anti-*T. gondii* activity against the RH strain *in vitro* and acute *in vivo* models. The inhibition of plaque formation, rapid alteration of extracellular tachyzoite morphology, suppression of parasite adhesion, invasion, and intracellular proliferation, together with the protective effects observed during acute infection, indicate that mangiferin exerts a broad antiparasitic phenotype rather than acting exclusively at a single stage of the parasite lytic cycle. These findings extend the evidence for the anti-*T. gondii* activity of mangiferin and provide a basis for further investigation of its mechanism of action, pharmacological properties, and potential therapeutic application.

## Data Availability

The original contributions presented in the study are included in the article/Supplementary material, further inquiries can be directed to the corresponding author/s.
