# Supplementary material for: Mangiferin inhibits multiple stages of the Toxoplasma gondii lytic cycle in vitro and protects against acute RH strain infection in vivo
Source: Front Microbiol. 2026 Oct 23;17:1983878. doi: 10.3389/fmicb.2026.1983878 (PMC13739697; doi:10.3389/fmicb.2026.1983878)
Supplement: Supplementary file 1 [file Table_1.DOCX]

Table S1. Primers used for qPCR

| Gene | Sequence (5′‑3′) |
| --- | --- |
| *SAG1* | F: ATCGCCTGAGAAGCATCACTG |
|  | R: CGAAAATGGAAACGTGACTGG |
| H-*Actin* | F: ATCAAGATCATTGCTCCTCCTGA |
|  | R: CTGCTTG CTGATCCACATCTG |
| M-*Actin* | F: CCTGTATGCCTCTGGTCGTA |
|  | R: CTGTGGTGGTGAAGCTGTAG |
